# Glucose metabolism in completed suicide: a forensic-pathological pilot study

**DOI:** 10.3325/cmj.2017.58.34

**Published:** 2017-02

**Authors:** Jonas Forsman, Terhi Keltanen, Benny Liberg, Antti Sajantila, Thomas Masterman, Katarina Lindroos

**Affiliations:** 1 Neuro division, Department of Clinical Neuroscience, Karolinska Institutet, Stockholm, Sweden; 2 Hjelt Institute, Department of Forensic Medicine, University of Helsinki, Helsinki, Finland; 3 Division of Medical Imaging and Technology, Department of Clinical Science, Intervention and Technology (CLINTEC), Karolinska Institutet, Stockholm, Sweden

## Abstract

**Aim:**

To determine whether antemortem blood levels of glycated hemoglobin (HbA_1c_) and glucose predict completed suicide and, by extension, whether markers of glucose metabolism might be associated with a prosuicidal trait or state.

**Method:**

From consecutively performed autopsies, samples of blood and vitreous humor from 17 suicide victims and 27 non-suicide controls were compared with regard to levels of glucose, lactate, and HbA_1c_.

**Results:**

Mean HbA1c was higher and mean estimated blood glucose was lower among suicide victims, although tests revealed no significant differences (*P* = 0.171 and *P* = 0.395, respectively). HbA1c levels exceeding 48.0 mmol/mol, which were indicative of persistent hyperglycemia, were twice as common in suicide victims (59% vs 30%; *P* = 0.068).

**Conclusion:**

The finding of this pilot study suggest that deranged glucose metabolism may reflect biological events antecedent to, or concomitant with, completed suicide, with the following clinical implications: recurring hyperglycemia due to defective glucose transport, which may give rise to depression and suicidal ideation, and elevated HbA_1c_ levels, which may represent an assayable correlate to neurobiological conditions predisposing to suicide.

Mood disorders are overrepresented in cases of completed suicide (1-4). Observational studies have shown that a diagnosis of diabetes mellitus – a condition characterized by persistent hyperglycemia – is associated with an increased risk of depression and anxiety in both adolescents and adults (5-10). In a recent cohort study of over 1.2 million Korean men and women, a single elevated fasting blood-glucose measurement was found to confer a two- to 3-fold increased risk of completed suicide during a 14-year follow-up (11), while a recent registry-based matched cohort study of the entire Swedish population demonstrated a 3.4-fold risk increase for suicide among subjects afflicted with diabetes (12). However, the mechanism by which deranged glucose metabolism could lead to suicide remains elusive.

Glucose is a ubiquitous source of energy, and under normal metabolic conditions—in the absence of fasting or other states of starvation—it is the human brain’s primary source of cellular fuel. Psychological processes such as self-control, decision making and regulation of emotions depend heavily on intracellular availability of glucose in the brain (13,14).

It has been suggested that dysregulation of emotions—resulting in aggressive impulses, pessimism and impulsivity—occurs more commonly among suicide attempters (15,16). Further, in males, aggressiveness and impulsivity have been shown to be associated to lower blood glucose levels (17). Although previous studies have identified several biochemical markers in completed suicide (18-20), to our knowledge no study has attempted to characterize the state of glucose metabolism in the weeks, hours and minutes prior to the suicidal act.

Hyperglycemia results from a reduced ability of cells to take up circulating glucose, on account of either insulin deficiency or insulin resistance. Elevated blood glucose levels can thus be interpreted as a sign of intracellular glucose deficiency. In the clinic, states of acute and chronic hyperglycemia are diagnosed by way of measurements in blood of glucose and glycosylated hemoglobin (HbA_1c_), respectively. While blood glucose reflects the current glycemic state, HbA_1c_—formed by non-enzymatic coupling of glucose and hemoglobin—reflects blood glucose levels over the past one to three months.

In the present pilot study, we compared subjects who had undergone forensic autopsy, grouped according to cause of death, to determine the extent to which glucose metabolism was deranged in the period preceding the suicidal act. We compared the estimated blood glucose and HbA_1c_ concentrations in cases of completed suicide and controls. In addition, we compared frequencies of subjects in each group whose HbA_1c_ concentrations exceeded the threshold for a diagnosis of diabetes mellitus at the time of autopsy.

## MATERIAL AND METHODS

### Subjects

Beginning in November 2012, in the course of 51 consecutive medico-legal autopsies performed at the Department of Forensic Medicine of the Hjelt Institute of the University of Helsinki, samples were collected post mortem according to standard procedures. All autopsies were conducted by the same forensic team, headed by a single pathologist.

At the time of autopsy, causes of death were determined and coded according to the International Statistical Classification of Disease, 10th revision (ICD-10). Subjects were divided into two groups according to ICD-10 codes: those who had died of suicide (ICD-10 codes X64-X84) and those who had died on account of other causes, including events of undetermined intent (ICD-10 codes Y10- Y34), who made up the control group.

Seven of the 51 subjects in original sample were excluded due to either protracted processes of postmortem decay, which made sampling impossible, or hemolysis of collected blood samples. Thus, a total of 44 individuals were included in the statistical analysis. Clinical and demographic data, as well as results of standard toxicological screening, were available for all 44 subjects.

### Samples

Samples of blood and vitreous humor were collected from the femoral vein and vitreous chamber according to standard forensic procedures. Levels of HbA_1c_ in blood were determined by high-pressure liquid chromatography using Mono-S cation-exchange columns, followed by conversion to the unit mmol/mol, as recommended by the International Federation of Clinical Chemistry (21).

Concentrations of glucose and lactic acid were determined by standard enzymatic assays, as described by Sippel and Möttönen (22). In accordance with formula of Traub, the sum of postmortem concentrations in vitreous humor of glucose and lactate was used to estimate antemortem glycemic state. Because levels of lactate in vitreous humor increase linearly with the duration of the postmortem interval (23), Traub-index levels were, for given individuals, not regarded as accurate estimates of antemortem blood glucose levels.

### Statistical analysis

Blood HbA_1c_ and Traub-index levels were compared in suicide victims and controls using Wilcoxon rank-sum test and Welch’s *t* test, respectively. In addition, frequencies of subjects in each group with blood HbA_1c_ levels exceeding the threshold for persistent hyperglycemia, as defined by the World Health Organization (24,25), were compared using Fisher’s exact test. Uncorrected *P* < 0.05 were regarded as statistically significant. All statistical analyses were performed using R (ver. 3.1.3) (26).

## RESULTS

The mean age at death and gender distribution were comparable between groups (Table 1). In the suicide group, physical trauma contributing to death was more common and pre-existing somatic illness less common. Antidepressants were detected in blood at similar rates in both groups. There were differences between groups with regard to the detection in postmortem blood of ethanol, as well as antidiabetic and antipsychotic medications — substances whose antemortem use may affect blood-glucose levels. With the exception of illicit drugs, all other analyzed substances, including alcohol, occurred more commonly in the non-suicide group.

**Table 1:** Demographic and toxicological data by cause of death in suicide vs non-suicide subjects

	No. (%) of subjects	
**Demographic characteristics**	**suicide (n = 17)**	**non-suicide (n = 27)**
Male	11 (65.0)	18 (67.0)
Age (years; mean±SD; median, range)*	55.0 ± 19.5 (63.5, 24-86)	59.3 ± 21.0 (58.3, 21-93)
Physical trauma involved	14 (82.0)	7 (26.0)
Known somatic illness	1 (6.0)	20 (74.0)
**Toxicological characteristics (number positive)**		
Ethanol	4 (24.0)	11 (41.0)
Benzodiazepines	5 (29.0)	11 (41.0)
Opiates	2 (12.0)	9 (33.0)
Other narcotic substances^†^	3 (18.0)	2 (7.0)
Antidepressants	4 (24.0)	8 (30.0)
Antipsychotics	1 (6.0)	5 (19.0)
Antidiabetic medications	0 (0.0)	2 (7.0)

*SD – standard deviation.

†Cannabis, amphetamine.

Of the 44 subjects, 17 were adjudged to have committed suicide and 27 to have died of other causes. Among the suicide victims, specific causes of death were hanging (n = 7), gunshot wounds (n = 5), poisoning (n = 3), exposure to fire (n = 1), and contact with a moving vehicle (n = 1). Among the non- suicide controls, specific causes of death were cardiovascular disease (n = 6), accidental poisoning (n = 6), falling accidents (n = 5), alcohol-related organ damage (n = 3), infectious disease (n = 2), homicide (n = 1), traffic accident (n = 1), Alzheimer disease (n = 1), and unknown causes (n = 2).

### Glucose

One subject in the non-suicide group displayed elevated ketone bodies in the absence of hyperglycemia and other findings indicative of alcohol-induced ketoacidosis. Mean values of Traub index, based on glucose and lactate measurements in the vitreous humor, did not differ significantly between the suicide and non-suicide group (35.3 ± 9.8 mmol/L vs 33.4 ± 4.9 mmol/L, respectively, *P* = 0.395; Table 2). Prior to analysis, the Shapiro-Wilk normality test had revealed that the values were normally distributed, allowing the use of a parametric test.

**Table 2:** Glycemic biomarkers by cause of death in suicide vs non-suicide subjects

	Bimarker concentration (mean±SD; median, range)*		
**Biomarker**	**suicide** (n = 17)	**non-suicide** (n = 27)	***P***
Vitreous glucose, mmol/L	0.9 ± 2.1 (0.0, 0.0-7.0)	1.5 ± 2.9 (0.0, 0.0-11.9)		
Vitreous lactate, mmol/L	32.5 ± 5.1 (32.4, 23.3-39.1)	33.9 ± 8.4 (34.9, 16.5-45.8)		
Vitreous Traub^†^, mmol/L	33.4 ± 4.9 (32.7; 23.3-41.1)	35.3 ± 9.8 (34.9, 17.2-56.7)	0.395^‡^	
Blood HbA_1c_, mmol/L	51.1 ± 14.7 (48.4, 35.6-97.8)	46.6 ± 13.8 (43.2, 21.9-99.6)	0.171^§^	
Blood HbA_1c_ (%)>48.0 mmol/mol (SEM)	59.0 (5.03)	30.0 (5.80)	0.068^‖^	

*SD = standard deviation; SEM = standard error of the mean.

†Formula of Traub = lactate in vitreous humor + glucose in vitreous humor.

‡Welch’s *t* test.

§Wilcoxon rank-sum test.

‖Fisher’s exact test.

### HbA_1c_

**Figure 1:**
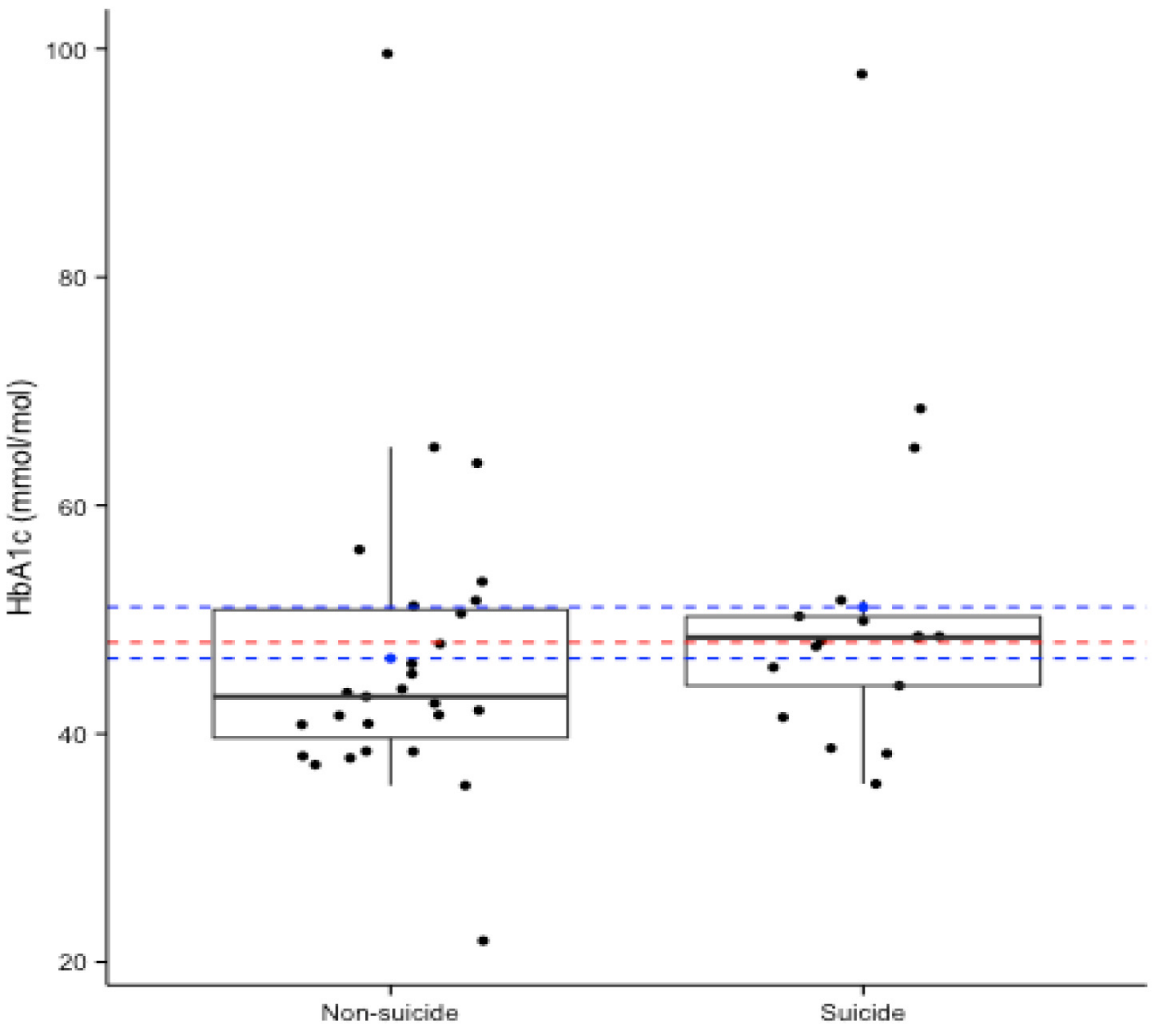
**HbA_1c_ levels in postmortem blood by cause of death in suicide vs non-suicide subjects.** The red dashed line indicates threshold recommended by the World Health Organization for diagnosis of diabetes mellitus (48.0 mmol/mol). The blue dashed lines indicate the mean value of each group. Groups did not differ significantly with regard to HbA_1c_ levels (*P* = 0.171); however, HbA_1c_ values exceeding 48.0 mmol/mol were twice as common in the suicide group (*P* = 0.068).

HbA_1c_ levels measured in postmortem blood were, on average, higher among suicide victims than among subjects who had died of other causes (51.1 ± 14.7 mmol/mol vs 46.6 ± 13.8 mmol/mol, respectively; Table 2); however, the Shapiro-Wilk normality test revealed that the values were not normally distributed, necessitating the use of a non-parametric test. When tested using the Wilcoxon rank-sum test, the groups did not differ significantly with regard to HbA_1c_ levels (*P* = 0.171; Table 2). In addition, HbA_1c_ values exceeding the WHO threshold for dysregulated glucose metabolism (48.0 mmol/mol, recommended for use in diagnosing diabetes mellitus ante mortem) were twice as common in the suicide group, compared to the non- suicide group (59% vs 30%, *P* = 0.068; Table 2 and Figure 1). The specific causes of death in subjects with HbA1c values exceeding 48.0 mmol/mol were, among suicide victims hanging (n = 4), gunshot wounds (n = 3), poisoning (n = 1), exposure to fire (n = 1) and contact with a moving vehicle (n = 1); and, among non-suicide controls, cardiovascular disease (n = 4), falling accidents (n = 2), accidental poisoning (n = 1) and traffic accident (n = 1).

## DISCUSSION

In the present study of glycemic biomarkers in consecutive forensic autopsies, ranked HbA_1c_ levels were found to be higher, albeit nonsignificantly, in subjects who had committed suicide than subjects who had died of other causes. Similarly, in two recent studies, HbA_1c_ levels were associated with suicidal ideation in subjects with either previously diagnosed (27) or undiagnosed (28) diabetes. The authors of the former study speculate that elevated HbA_1c_ levels may reflect poor self-care associated with depression; whereas the authors of the latter study propose that defective glucose transport, resulting in recurring hyperglycemia, in itself may directly give rise to depression and suicidal ideation–may in other words, constitute a persisting, prosuicidal “trait.”

In the present study, we have also investigated whether glucose metabolism might play a role in the prosuicidal “state” – the suicidal act’s concomitant neurobiological substrate. We found that estimated antemortem blood glucose levels were slightly lower in subjects who had committed suicide than in subjects who had died of other causes, including somatic illness – although again the difference was not statistically significant. In the present data set, however, the extent to which the duration of antemortem agony differed between the two groups is unclear. Antemortem agony is, of course, associated with the release of stress hormones resulting in, among other things, transient elevation of blood glucose. By the same token, the reliability of approximations of antemortem blood glucose concentration, using Traub’s formula, is inversely proportionate to the postmortem interval (23), which, in Finland, as has been previously reported, is, on average, relatively long (29). Indeed, in a coming study in a large Swedish autopsy series, we hope to restrict our analysis of estimated blood glucose levels to cases of immediate death.
